# Comparison of the efficacy of *Aeromonas veronii* Δ*hisJ* vaccine in *Carassius auratus* via different immunization routes

**DOI:** 10.3389/fvets.2024.1378448

**Published:** 2024-03-21

**Authors:** Tonglei Wu, Ruitao Ma, Xiaoyi Pan, Fengjie Wang, Zhiqiang Zhang, Qiumei Shi, Xiaofeng Shan, Guisheng Gao

**Affiliations:** ^1^College of Animal Science and Technology, Hebei Normal University of Science & Technology, Qinhuangdao, China; ^2^College of Animal Science and Technology, Jilin Agricultural University, Changchun, China; ^3^Zhejiang Institute of Freshwater Fisheries, Huzhou, China

**Keywords:** *Aeromonas veronii*, *hisJ*, vaccine, *Carassius auratus*, immune route

## Abstract

**Introdction:**

*Aeromonas veronii* is a significant pathogen to various aquatic life. Infections in fish can lead to high mortality rates, causing substantial economic losses in aquaculture. Vaccination is proposed as a substitute for antibiotics in aquaculture to decrease disease-related mortality and morbidity. Our study previously constructed a *hisJ*-deleted strain of *A. veronii*, which provided protective effect to *Loach*.

**Methods:**

To further assess the vaccine’s applicability, this study evaluated its genetic stability and safety, and the immune protective effects in *Carassius auratus* through four distinct administration routes: intraperitoneal injection, intramuscular injection, oral administration, and immersion, to determine the efficacy of these administration routes.

**Results:**

The results showed that the vaccine remained genetically stable after 45 generations. Immunization via these administration routes was safe for *Carassius auratus*, with intraperitoneal and intramuscular injections causing stronger adverse reactions. Immersion immunization resulted in mild adverse reactions, and no significant adverse reactions were observed following oral immunization. Immunizing *Carassius auratus* at safe concentrations via these routes enhanced the phagocytic activity in serum, increased the levels of non-specific immune-related enzymes (ACP, AKP, C3, C4, LZM, SOD, and IgM), and improved specific serum antibody levels. It also elevated levels of cytokines related to inflammatory responses (IL-1β, IL-10, TNF-α, TGF-β) in organ tissues (liver, spleen, kidney, mid-post intestine, and gills). The survival rates of *Carassius auratus* were measured after challenging with the virulent strain *A. veronii TH0426*, resulting in the relative survival rates of 64% for Intraperitoneal vaccine group, 56% for Intramuscular vaccine group, 52% for oral vaccine group, and 48% for immersion vaccine group. Analysis of bacterial load in the liver, spleen, and kidney post-challenge showed a decreasing trend in the control group, indicating that the vaccine strain *ΔhisJ* could gradually restrict the rapid proliferation of bacteria in these tissues, thereby providing a certain level of immune protection against *A. veronii*.

**Discussion:**

In brief, the vaccine strain *ΔhisJ* can serve as a safe live attenuated vaccine for *Carassius auratus*, and this study lays the foundation for the development of live attenuated vaccines against *Aeromonas veronii*.

## 1 Introduction

*Carassius auratus*, a term encompassing varieties like *Goldfish*, Fangzheng *Carassius auratus*, Pengze *Carassius auratus*, and Polyploid *Carassius auratus*, is a species with a wide range of varieties (1, 2). It is a historically significant freshwater cultured fish in China and East Asia. Due to its delicious taste and high economic value, it is favored by aquaculturists and has a huge market demand (3). With the widespread popularization of intensive farming, bacterial diseases in *Carassius auratus* have become frequent, among which *Aeromonas* is the most common bacterial pathogen. *Aeromonas* is widely distributed in freshwater and sewage environments and can also be isolated from food, such as meat, aquatic products, and milk, posing certain hazards to humans and aquatic animals (4–6). *Aeromonas veronii* (*A.veronii*), one of the main pathogens in the *Aeromonas* genus, can infect many species of freshwater and marine fish, including *Carassius auratus*, *Tilapia*, *Catfish*, *Largemouth bass*, *Loach*, *Salmon*, and *Turbot*, as well as *Crustaceans* and *Mollusks* (7–9). Clinical symptoms of infection mainly include septicemia, gill rot, enteritis, skin ulcers, and visceral infections. In aquaculture, *A.veronii* infection can lead to high mortality rates, causing significant economic losses.

Antibiotic treatment is the preferred method to control bacterial infections, providing good preventive and therapeutic effects, but also brings many challenges, such as antibiotic residues and emergence of multi drug-resistant strains, which are passed to humans through drinking water and food, posing serious threats to human health. Vaccine immunization is another important measure to prevent and control bacterial infections. In aquaculture, vaccines mainly include attenuated live vaccines, inactivated vaccines, subunit vaccines, DNA and mRNA vaccines (10–12). Among them, the live attenuated vaccines can simulate the natural infection process, stimulating innate immunity, cellular immunity, and specific immunity, and have been proven to provide more effective and lasting immune protection. Using genetic engineering technology to knock out the virulence genes is a popular method for preparing live attenuated vaccines. Researchers prepared a live attenuated *A.veronii* vaccine by suppressing the expression of SmpB, to immunize *Zebrafish*, and achieved a survival rate of 65% after challenge with a virulent strain (13). A *tolC* gene-deleted live attenuated vaccine significantly reduced tissue damage in *Hyriopsis cumingii* infected by a virulent strain (14). An *hcp* gene-deleted attenuated vaccine immunized the largemouth bass, achieving a 100% survival rate, significantly higher than the 76.67% survival rate of the formalin inactivated vaccine immunization group (15). Our research group prepared a live attenuated vaccine by knocking out the *hisJ* gene, which enabled a survival rate of 65.66% in the *Loach* against the virulent strain (16). Despite these advancements, numerous challenges persist. The safety of the live attenuated vaccines raises concerns, particularly regarding their potential reversion to virulence or adverse effects in immunocompromised individuals. And, the precise duration of immunity these vaccines confer remains elusive, prompting inquiries into the necessity for booster shots and sustained protection for vaccinated groups (13–15, 17). Therefore, while *A.veronii* vaccine development is a promising strategy for infection control, it necessitates ongoing research to address these prevailing issues.

The immunization pathway is an important factor determining the vaccine’s immune protective effect. The administration routes of fish vaccine include injection, oral, and immersion. Injection immunization includes muscle injection and intraperitoneal injection (14, 18). The site for muscle injection is generally the muscle at the base of the dorsal fin or tail fin of the fish, while the intraperitoneal injection site is often inside the peritoneum at the base of the ventral fin. The injection route produces strong immune protection and long-lasting immunity, but the injection process is time-consuming, labor-intensive, and highly stressful for the fish. The oral immunization route involves mixing the vaccine with feed and administering it orally when the fish feed, saving a lot of manpower and avoiding the stress and irreversible damage caused to the fish during the injection process. However, the oral immunization method still requires fishing operation, uses a large amount of vaccine, and vaccine components are easily degraded by gastrointestinal proteases, losing immunogenicity, resulting in a relatively poor immune protection effect (19). Immersion immunization involves immersing fish in water containing the vaccine for a period, which requires less manpower, allows immunization during transport, causes minimal fish injury, and can even avoid fishing during immunization. However, immersion immunization consumes a large amount of vaccine and often provides low protection and short immunity duration (20).

Our research group’s earlier construction of an *hisJ*-deleted vaccine of *A.veronii* (namely *ΔhisJ*) achieved good immune protection in the *Loach* (16, 21). Genetic stability, virulence, and immune protective efficacy are key indicators for evaluating live attenuated vaccines. To analyze the immunological effects of different administration routes on *Carassius auratus* and further evaluate its potential as a live attenuated vaccine, this study utilized passage experiments to assess the genetic stability of the live attenuated vaccine *ΔhisJ*. The safety evaluating results of the vaccine showed that intraperitoneal and intramuscular injection of the vaccine caused inflammatory reactions at the injection site, while oral and immersion immunization routes did not result in significant stress response. The relative survival rates were obtained as 64% in the IpV group, 56% in the ImV group, 52% in the OV group, and 48% in the IV group. The results laid a foundation for the development of a live attenuated vaccine *ΔhisJ*.

## 2 Materials and methods

### 2.1 Rearing of *Carassius auratus*

The experimental *Carassius auratus* were purchased from a fish farm in Changchun, with each fish weighing an average of 50 ± 5 g. After disinfection of the fish surfaces through immersion in potassium permanganate and saline solution, they were temporarily housed in aquarium tanks in the Aquaculture Laboratory of Jilin Agricultural University. Consistent oxygen supply and temperature were maintained in the tanks, with one-third of the water being changed every 2 ds. Once the fish had stable growth and adapted to the water environment, they were randomly assigned to groups for bacterial infection and immunity experiments.

### 2.2 Strains and cultural methods

In earlier studies, a live attenuated vaccine strain, designated as *ΔhisJ*, was developed by knocking out the *hisJ* gene in the wild type *A.veronii TH0426* (GenBank accession no. CP012504.1) (21).

The wild-type *A.veronii TH0426* (which are Ampicillin-resistant) and the attenuated vaccine strain *ΔhisJ*, preserved in the laboratory, were streaked onto RS agar plates (Hopebiol, Qingdao, China) and incubated at 30°C for 12 h. Single colonies were selected and inoculated into liquid LB medium (containing 0.1% Ampicillin), then cultured at 30°C with agitation at 180 rpm for 12 h. The bacterial culture was subsequently centrifuged at 6000 rpm for 5 min; the supernatant was discarded, and the pellet was washed three times with PBS and resuspended in the same volume. Finally, 25% glycerol was added, and the mixture was thoroughly mixed before being stored at −80°C in an ultra-low-temperature refrigerator. The next day, a portion of the frozen bacterial culture was retrieved and counted using a serial dilution technique. The process involves diluting a bacteria sample several times to break the sample into manageable concentration levels. After each dilution, a portion of the sample may be plated onto a LB agar plate, and after incubation, the colonies are counted. This step was crucial as it preceded the quantitative determination of the average colony-forming units per milliliter (CFU/mL), a critical measure determining the bacterial concentration used for subsequent challenge and vaccinations.

Employing the method outlined above, *Staphylococcus aureus CMCC(B)26,003* was cultivated and enumerated, with the culture conditions maintained at a temperature of 37°C. The bacterial culture was then adjusted to a concentration of 1 × 10^7^ CFU/mL, suitable for subsequent phagocytosis assays.

### 2.3 Verification of the genetic stability of the vaccine strain *ΔhisJ*

The vaccine strain *ΔhisJ* is being tested for genetic stability over multiple generations of growth in LB medium, using PCR amplification. The strain *ΔhisJ* was continuously passaged for 45 generations in LB liquid medium containing 0.1% Ampicillin, with genome extraction every 5 generations. At the same time, PCR identification was performed using the primers *hisJ*-for/*hisJ*-rev (Table 1). The target bands were sent to Kumei (Changchun) Biotech Co., Ltd. for sequencing. The purpose of the experiment is to confirm that the mutant remains consistent over many generations, ensuring the strain’s stability for any application in vaccine development.

**Table 1:** Primers in this study.

Primers	Sequence (5′-3′)
*hisJ*-for	CGTCTAGAGCTTACCTCGTCTTGATGGCA
*hisJ*-rev	TAGAGCTCGCTGAAGGTTGCCATCGGTTA
IL-10-for	GGAACGATGGGCAGATCAA
IL-10-rev	AACTGAAGGGGAAGGGGA
IL-1β-for	AACTGATGACCCGAATGGAAAC
IL-1β-rev	CACCTTCTCCCAGTCGTCAAA
TNF-α-for	TTATGTCGGTGCGGCCTTC
TNF-α-rev	AGGTCTTTCCGTTGTCGCTT
TGF-β-for	CTGGCTCTTGCTCTTTCGTCT
TGF-β-rev	AAGGATGGGCAGTGGGTCT
β-actin-for	CAAGATGATGGTGTGCCAAGTG
β-actin-rev	TCTGTCTCCGGCACGAAGT

### 2.4 Determination of the median lethal dose (LD_50_) of the *Carassius auratus*

To determine the optimal immune dosage for immunizing *Carassius auratus* and confronting them with a bacterial challenge, we employed various administration routes for the wild-type strain *A. veronii TH0426* and the live attenuated vaccine *ΔhisJ*, including intraperitoneal and muscular injections, along with immersion and oral routes. A total of 120 *Carassius auratus*, evenly divided into 8 groups, were immunized with different types and doses of vaccines. Over a period of 14 ds, meticulous daily monitoring was conducted to record the mortality rates of *Carassius auratus*. Subsequently, these data were utilized to calculate the LD_50_ for the respective bacterial strains employing the modified Karber method. The calculation formula is as follows:

LD_50_ = log-1[Xm − *i*(*p* − 0.5)], note: *i* is the dose interval (i.e., the difference between the logarithmic doses of two adjacent dose groups); Xm is the logarithm of the maximum dose; *p* is the mortality rate of each dose group (mortality rates are all expressed in decimals); *p* is the sum of the mortality rates of each dose group.

Different types of vaccines are prepared according to the methods described below. Both strains, *TH0426* and *ΔhisJ*, were initially cultured in LB medium before being washed and resuspended in PBS. *TH0426* was administered through intraperitoneal injection, each injection consisting of 200 μL of bacterial solution at concentrations ranging from 2 × 10^8^ to 2 × 10^4^ CFU/mL. In contrast, *ΔhisJ* was injected either intraperitoneally or into the muscle near the dorsal fin, with each injection being 200 μL and concentrations varying from 2 × 10^9^ to 2 × 10^5^ CFU/mL. During the immersion tests, the *TH0426* and *ΔhisJ* culture was added to the aquarium, with the fish being immersed for 30 min in solutions containing bacterial concentrations ranging from 2 × 10^8^ to 2 × 10^4^ CFU/mL, and from 2 × 10^9^ to 2 × 10^5^ CFU/mL. Following this treatment, the fish were relocated to a bacteria-free aquarium for subsequent rearing. For the oral tests, the concentration of *ΔhisJ* was adjusted to levels ranging from 2 × 10^8^ to 2 × 10^4^ CFU/mL, and from 2 × 10^9^ to 2 × 10^5^ CFU/mL, respectively. Every 200 μL of this bacterial solution was combined with 100 g of fish feed (supplied by TONGWEI Co., LTD., China). Additionally, to ensure the bacteria were adequately encapsulated, 3 g of sodium alginate powder (sourced from Sigma-Aldrich, United States) was used at a 1.5% ratio during the preparation of the fish feed. After the encapsulation process, which involved drying, the feed was refrigerated at 4°C and preserved for future feeding sessions.

### 2.5 Preparation of different types of vaccines

For vaccines administered by intraperitoneal (IpV group) or intramuscular injection (ImV group), the concentration of the attenuated vaccine strain *ΔhisJ* is 2 × 10^7^ CFU/mL. For vaccines used in immersion immunization (IV group), the culture of *ΔhisJ* is added to the aquarium to achieve a concentration of 2 × 10^7^ CFU/mL. For vaccines used in oral immunization (OV group), the concentration of the *ΔhisJ* is adjusted to 2 × 10^7^ CFU/mL, and for every 200 μL of bacterial solution, it is mixed with 100 g of fish feed.

### 2.6 Animal safety assessment of vaccine

To assess the safety of the attenuated vaccine strain *ΔhisJ*, the *Carassius auratus* were randomly divided into five groups, with 10 fish in each group, for a challenge test. For the IpV group and the ImV group, the dose of the vaccine was 200 μL per fish. For the IV group, the *Carassius auratus* were first soaked in an aquarium containing the attenuated vaccine for 30 min, and after the immunization, the fish were transferred to a vaccine-free aquarium. For the OV group, fish were fed vaccine-coated feed at a dose of 2% of body weight, continuously for 5 ds, and after the immunization, they were fed non-vaccine-coated feed. After the immunization, continuous observation was carried out for 1 week, and the clinical symptoms and mortality rate of *Carassius auratus* in each group were recorded. *Carassius auratus* were intraperitoneally and intramuscular injected with an equal volume of PBS as a control group (namely Control group).

### 2.7 Vaccine immunization

*Carassius auratus* were divided into 7 groups, each consisting of 60 fish. In the intraperitoneal vaccine group (IpV group) and the intramuscular vaccine group (ImV group), the fish were subjected to either intraperitoneal injections or dorsal fin muscle injections of the attenuated vaccine strain *ΔhisJ*. A second dose of the vaccine was administered on the 14th d, following the same procedures. For the immersion vaccination group (IV group), the fish were immersed in an aquarium with a 2 × 10^7^ CFU/mL concentration of the vaccine. After soaking for 30 min, they were transferred to a vaccine-free aquarium. A second round of immersion vaccination was conducted 14 ds later. In the oral vaccine group (OV group), each fish received vaccine-coated feed amounting to 2% of its body weight. This method of oral immunization continued for 5 ds, followed by another 5 ds session starting on the 14th day. After immunization, the fish were given feed not coated with the vaccine.

For both the intraperitoneal PBS group (IpP group) and the intramuscular PBS group (ImP group), the *Carassius auratus* were injected with 0.2 mL of PBS per fish, either intraperitoneally or in the dorsal fin muscle, with a follow-up injection on the 14th d. Furthermore, a Blank Control group (BC group) was established in which the fish did not receive any form of vaccine immunization or PBS injections.

### 2.8 Sample collection

For serum samples, blood was drawn from the caudal vein of 10 randomly chosen healthy *Carassius auratus* on the 0, 7th, 14th, 21st and 28th d post immunization. The collection was performed post-anesthesia. Half of the obtained blood was treated with a sodium heparin solution (heparin sodium 1,000 IU/mg in 0.85% NaCl) to serve as an anticoagulant, intended for subsequent leukocyte phagocytic activity analysis. The remaining blood was subjected to centrifugation at 3000 rpm for 15 min to separate the supernatant, earmarked for evaluating various serum immune indices and antibody levels.

As for tissue samples, after the blood collection, the same fish were dissected to harvest specific organs and tissues, including the liver, kidneys, spleen, intestines, and gills. These specimens were promptly stored in a −80°C freezer, preserving them for future RNA extraction processes and cytokine assays.

### 2.9 Detection of non-specific immune indicators in serum

According to the manufacturer’s instructions, we utilized ELISA kits from the Nanjing Jiancheng Bioengineering Institute, Jiangsu, China, to analyze the enzyme activities of acid phosphatase (ACP), alkaline phosphatase (AKP), superoxide dismutase (SOD), and lysozyme (LZM). One Ginoff unit of AKP and ACP activity corresponds to 1 mg of phenol produced in 100 mL of serum reacting with the substrate at 37°C for 30 min, with the absorbance measured at 520 nm. One unit of SOD activity refers to the amount of enzyme that results in a 50% inhibition rate of SOD in the reaction system, measured by the absorbance at 450 nm. One unit of LZM activity was defined as the amount of lysozyme that causes a 0.001 unit reduction in absorbance per minute at 530 nm. All analyses were conducted in triplicate.

Based on the instruction manuals of the fish IgM ELISA kit (Nanjing Jiancheng Bioengineering Institute, Jiangsu, China), and the fish complement proteins C3 and C4 ELISA kits (Yan Domain Chemical Reagent Co., Ltd., Shanghai, China), the levels of IgM antibodies, C3 and C4 in serum were measured using a double-antibody sandwich ELISA method. The coating micropores provided in these kits were pre-coated with mouse anti-carp IgM, complement C3, and complement C4 antibodies. Standards, serum samples, and horseradish peroxidase (HRP)-labeled detection antibodies were added sequentially to the wells of the ELISA plate, followed by incubation and washing. Subsequently, tetramethylbenzidine (TMB) substrate solution was added for color development, and the reaction was stopped with H_2_SO_4_. The optical density (OD) was measured using a spectrophotometer at a wavelength of 450 nm. This value positively correlated with the corresponding antibodies in the sample. The concentration of complement C3 and C4 in the samples was calculated using a standard curve. All analyses were conducted in triplicate.

### 2.10 Serum specific antibody detection

Using a 96-well V-bottom plate to determine the agglutination titer of serum, the level of specific antibodies is evaluated. The specific procedure is as follows:

*A.veronii TH0426* is cultured until it reaches the logarithmic phase. After centrifugation, it is washed three times with PBS, and the bacterial suspension’s concentration is adjusted to 1 × 10^7^ CFU/mL. The bacteria are then inactivated by heating at 56°C for 30 min, serving as the inactivated bacterial antigen for the test. Take a 96-well plate and add 80 μL of saline solution to the first well, and 50 μL of saline solution to the remaining wells. Then add 20 μL of serum to the first well. Mix it evenly, then transfer 50 μL from this well to the second well. Using the same method, continue the process until the penultimate seventh well, discarding 50 μL each time. This creates a series of dilutions of the serum antibodies in the wells at 1:4, 1:8, 1:16, 1:32, 1:64, 1:128, 1:256, and 1:512 concentrations. The eighth well functions as a control and does not contain any serum. Distribute 50 μL of the deactivated bacterial antigen into every well, ensuring thorough mixing. Subsequently, allow the plate to incubate at 37°C for a duration of 2 h, and then let it remain at ambient temperature throughout the night. In the final step, assess the extent of bacterial agglutination through microscopic examination.

### 2.11 Detection of white blood cell phagocytic activity

*Staphylococcus aureus CMCC(B)26,003* was inoculated into TSB medium (Hopebio, Qingdao, Shandong, China) and subsequently washed three times with PBS. The bacterial suspension was then adjusted to a concentration of 1 × 10^7^ CFU/mL, used for phagocytic antigen. We then mixed 0.2 mL of anticoagulated blood with 0.02 mL of the prepared phagocytic antigen. This mixture was incubated in a water bath set at 25°C for 60 min, with gentle agitation every 10 min to ensure uniform consistency. After incubation, the mixture was centrifuged at 4000 rpm for 10 min, resulting in the formation of distinct layers. We carefully pipetted the intermediate layer enriched with white blood cells, depositing 5–10 μL droplets onto a glass slide to create a blood smear. The smear was allowed to air-dry and was then secured for the Wright-Giemsa staining process. We applied the stain to the blood smear for 10 min. Any residual stain was removed with distilled water, and the slide was left to dry completely. For the analysis, the slide was positioned under a microscope. We chose a random field of vision to scrutinize the white blood cells, evaluating their phagocytic activity towards bacteria. This observation was pivotal for determining the Phagocytic Percentage (PP) of white blood cells using the formula Equation 1:


(1) $$PP=\left(\begin{array}{l} \mathrm{Number}\;\mathrm{of}\;\mathrm{white}\;\mathrm{blood}\;\mathrm{cells}\\ \mathrm{phagocytizing}\;\mathrm{bacteria}/\\ \mathrm{Total}\;\mathrm{number}\;\mathrm{of}\;\mathrm{white}\;\mathrm{blood}\;\mathrm{cells} \end{array}\right)\times 100\%$$

### 2.12 Extraction of RNA and qRT-PCR analysis of cytokines

RNA was extracted from the liver, kidneys, spleen, intestines, and gills of *Carassius auratus* following vaccination, according to the instructions of the RNA extraction kit (TaKaRa, Beijing, China). Reverse transcription was carried out using a reverse transcription kit (TaKaRa, Beijing, China) to obtain cDNA. The cDNA obtained from reverse transcription served as a template for qPCR, performed with the TB Green^®^ Premix Ex Taq^™^ II kit (TaKaRa, Beijing, China). The primer sequences for each cytokine are presented in Table 1. RNA-free water was added for the negative control, and β-actin was used as the internal reference gene. The relative expression levels of each gene were calculated using the 2^−ΔΔCT^ method. All qRT-PCR reactions were set up in triplicate.

### 2.13 The challenge test by the wild type strain *A.veronii TH0426*

On the 35th d after the first immunization, 25 fish from each group were randomly selected for the challenge test by intraperitoneal injection of *A.veronii TH0426*. The immune protective effect in each experimental group was observed. *A.veronii TH0426* was revived and, after bacterial counting, the concentration was adjusted to 1.2 × 10^7^ CFU/mL to serve as the challenge bacterial solution. Each *Carassius auratus* was injected intraperitoneally with a dose of 0.2 mL. The experiment and control groups were observed for 14 ds, and any dead fish were promptly removed and recorded.

The relative percent survival for each group was calculated based on the mortality rates using the formula:


$$\begin{array}{l} \mathrm{Relative}\;\mathrm{Percent}\;\mathrm{Survival}=\\ \left(\begin{array}{l} 1-\mathrm{mortality}\;\mathrm{rate}\;\mathrm{in}\;\mathrm{immunized}\;\mathrm{group}/\\ \mathrm{mortality}\;\mathrm{rate}\;\mathrm{in}\;\mathrm{control}\;\mathrm{group} \end{array}\right)\times 100\%\text{.} \end{array}$$

### 2.14 Determination of bacterial load in tissues after challenge

On the 1st, 3rd, and 5th d following the challenge, liver, spleen, and kidney tissues were collected from moribund fish in each group as samples for testing. The organs were weighed, and then 0.4 g of tissue from each organ was randomly cut and placed into an EP tube containing 4 mL of sterile PBS. After mincing, the tissues were homogenized, and the homogenate was diluted in a 10-fold gradient and then spread onto RS agar plates. Following overnight incubation at 37°C, colony counting was conducted. The bacterial load in the liver, spleen, and kidneys at different time intervals for each experimental group was calculated to assess the immune protection efficacy of the vaccine.

### 2.15 Statistical analysis

Statistical analyses were conducted using SPSS 26.0 and GraphPad Prism v9.5.0, employing one-way Analysis of Variance (ANOVA) followed by *t*-tests. Data are presented as mean ± standard error. Significant differences are indicated with an asterisk (*), where *p < 0.05, **p < 0.01, and ***p < 0.001 are considered to represent statistically significant differences in mean values.

## 3 Results

### 3.1 Identification of genetic stability in the vaccine strain *ΔhisJ*

**Figure 1:**
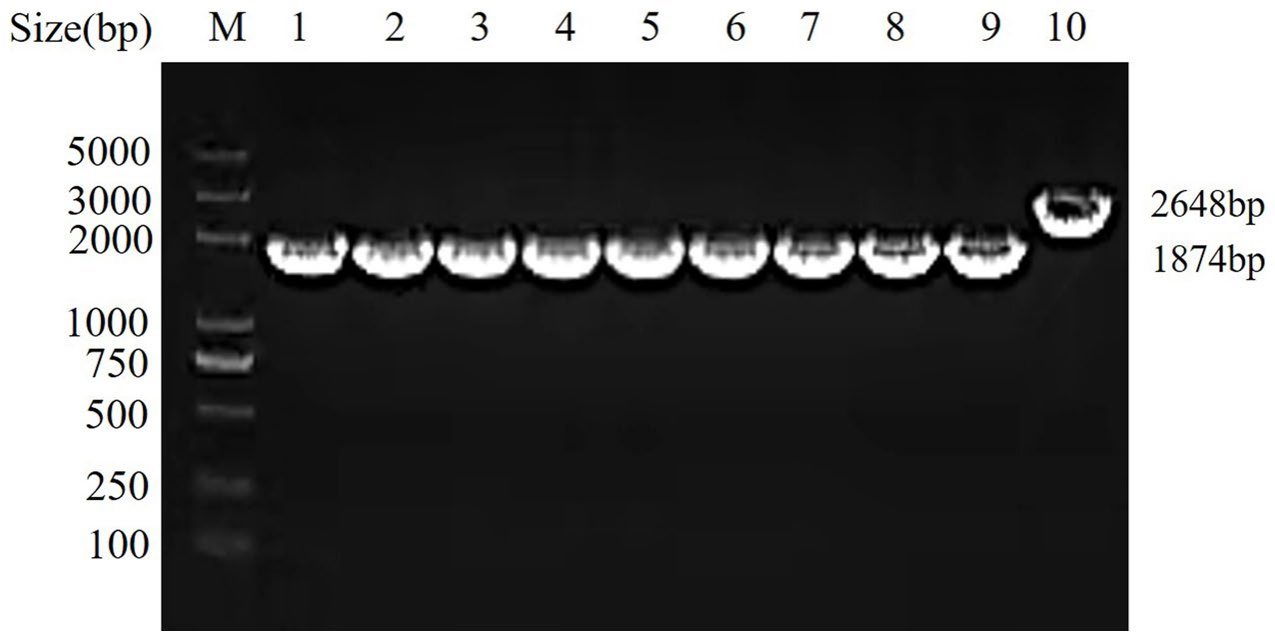
Genetic stability test of the vaccine strain *ΔhisJ.* M: DL2000 DNA Marker; 1–9: the vaccine strain *ΔhisJ*: a PCR verification is performed after every five passages; 10: the wild type strain *A.veronii TH0426.*

To assess the genetic stability of the *hisJ-*deleted strain, the *ΔhisJ* was serially passaged for 45 generations, and PCR amplification was conducted using the primers *hisJ*-for and *hisJ*-rev. In the wild type strain *A.veronii TH0426*, a 2,648 bp target band (including the ORF of *hisJ*) was amplified. In the *ΔhisJ* across different generations, a consistent 1874 bp target band was amplified, as shown in Figure 1. This result aligns with the expectations, demonstrating that the *hisJ*-deleted strain has good genetic stability.

### 3.2 Median lethal dose

To determine the immunization and the challenge dose, the LD_50_ of *A.veronii TH0426* and *ΔhisJ*. The results (Table 2) showed that under different administration routes, the LD_50_ of *ΔhisJ* significantly increased compared to the wild type strain *TH0426*. Specifically, in the IpV group, the LD_50_ increased by 357.8 times (2.10 × 10^8^/5.87 × 10^5^), the largest increase, while in the IV group, it increased by 146.9 times (2.16 × 10^8^/1.47 × 10^5^), the smallest increase. Across the four inoculation routes, the highest bacterial concentration at which *Carassius auratus* did not die when inoculated with *ΔhisJ* was 2 × 10^7^ CFU/mL. The maximum LD_50_ for the wild-type strain *TH0426* was 3.55 × 10^6^ CFU/tail. It is noteworthy that under the IpV injection route, the LD_50_ for fish was the smallest, indicating that intraperitoneal injection is the most virulent route for the strain in fish. Therefore, this study chose a dose of 4 LD_50_ (≈2.4 × 10^6^ CFU/tail) for intraperitoneal injection to conduct immune protection experiments. This involved adjusting the concentration of *ΔhisJ* to 2 × 10^7^ CFU/mL with an immunization dose of 0.2 mL, and adjusting the concentration of *TH0426* to 1.2 × 10^7^ CFU/mL with a virulent dose of 0.2 mL. The clinical symptoms exhibited by *Carassius auratus* infected with the wild-type strain *A.veronii TH0426* and the vaccine *ΔhisJ* included darkening of the body, slowed swimming, floating to the surface, surface bleeding, abdominal swelling, and in severe cases, skin ulcers.

**Table 2:** LD_50_ for different routes of exposure.

Immune route	LD_50_ (unit:CFU/tail)	
	*TH0426*	*ΔhisJ*
IpV	5.87 × 10^5^	2.10 × 10^8^
ImV	1.30 × 10^6^	3.06 × 10^8^
IV	1.47 × 10^6^	2.16 × 10^8^
OV	3.55 × 10^6^	5.32 × 10^8^

### 3.3 Animal safety test

**Figure 2:**
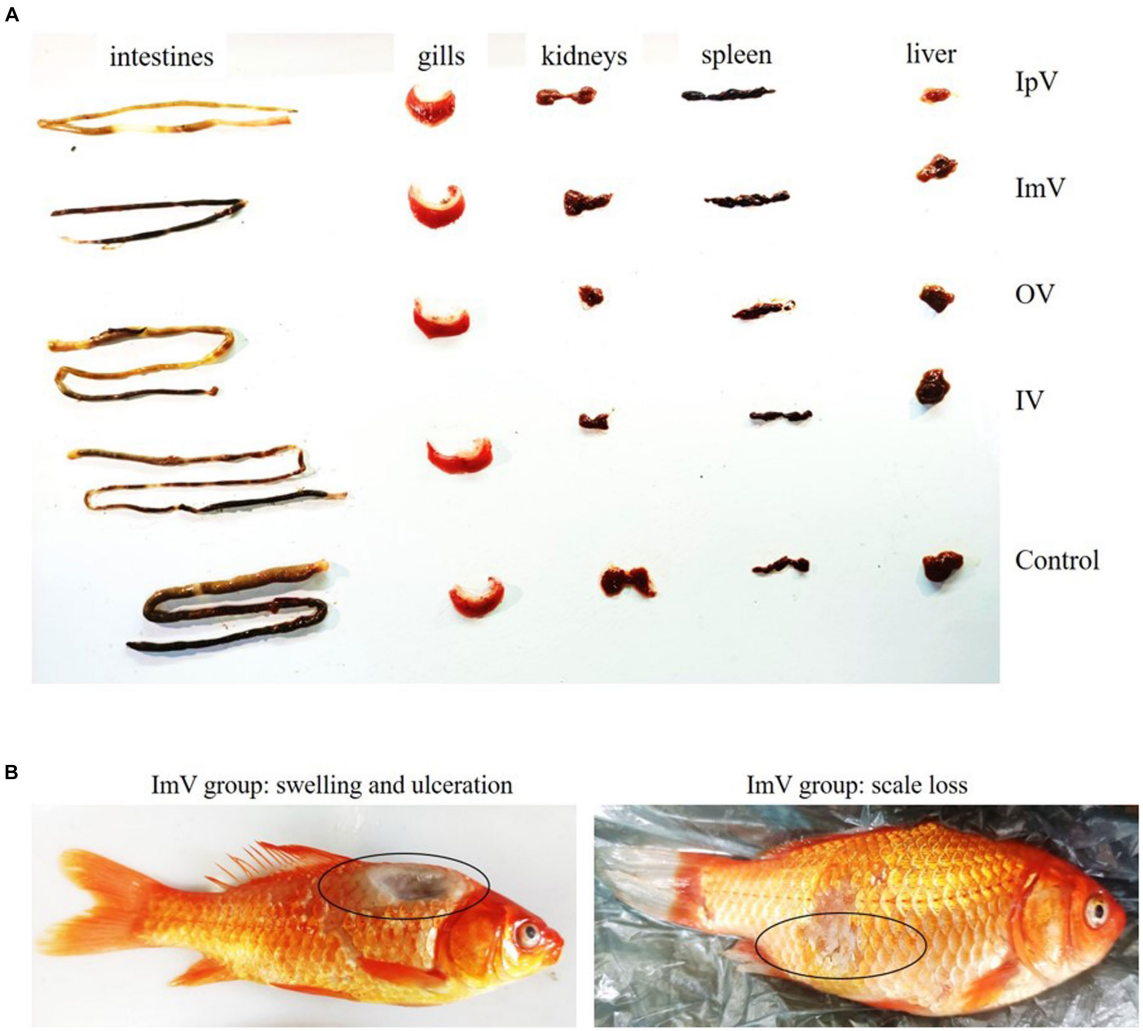
The animal safety evaluation of the vaccine strain Δ*hisJ*. **(A)** Organ diagram of *Carassius auratus* in the immunized and control group. The organs of the fish in each group did not show significant pathological changes. **(B)** In the ImV group, there was scale loss, swelling, and ulceration at the injection site.

In the groups of *Carassius auratus* immunized via intraperitoneal vaccination (IpV), intramuscular vaccination (ImV), immersion vaccination (IV), oral vaccination (OV), and intraperitoneal PBS injection (IpP), there were no instances of death following the administration of the vaccine or PBS. Fish in the IpV and ImV, groups exhibited strong stress responses, showing signs of poor mental state, reduced feeding, slow swimming, and floating to the water surface, but they returned to normal the next day. Fish in the IV group showed mild stress responses, with a slight reduction in feeding, but recovered to normal 6 h after immunization. Fish in the OV group did not show any stress responses. Upon dissection of fish from each group, there were no obvious pathological changes observed in the intestines, gills, kidneys, spleen, or liver of any group (Figure 2A). Notably, some fish in the ImV group experienced partial scale loss and even swelling and ulceration at the injection site (Figure 2B).

### 3.4 Nonspecific immune-related analysis in serum

**Figure 3:**
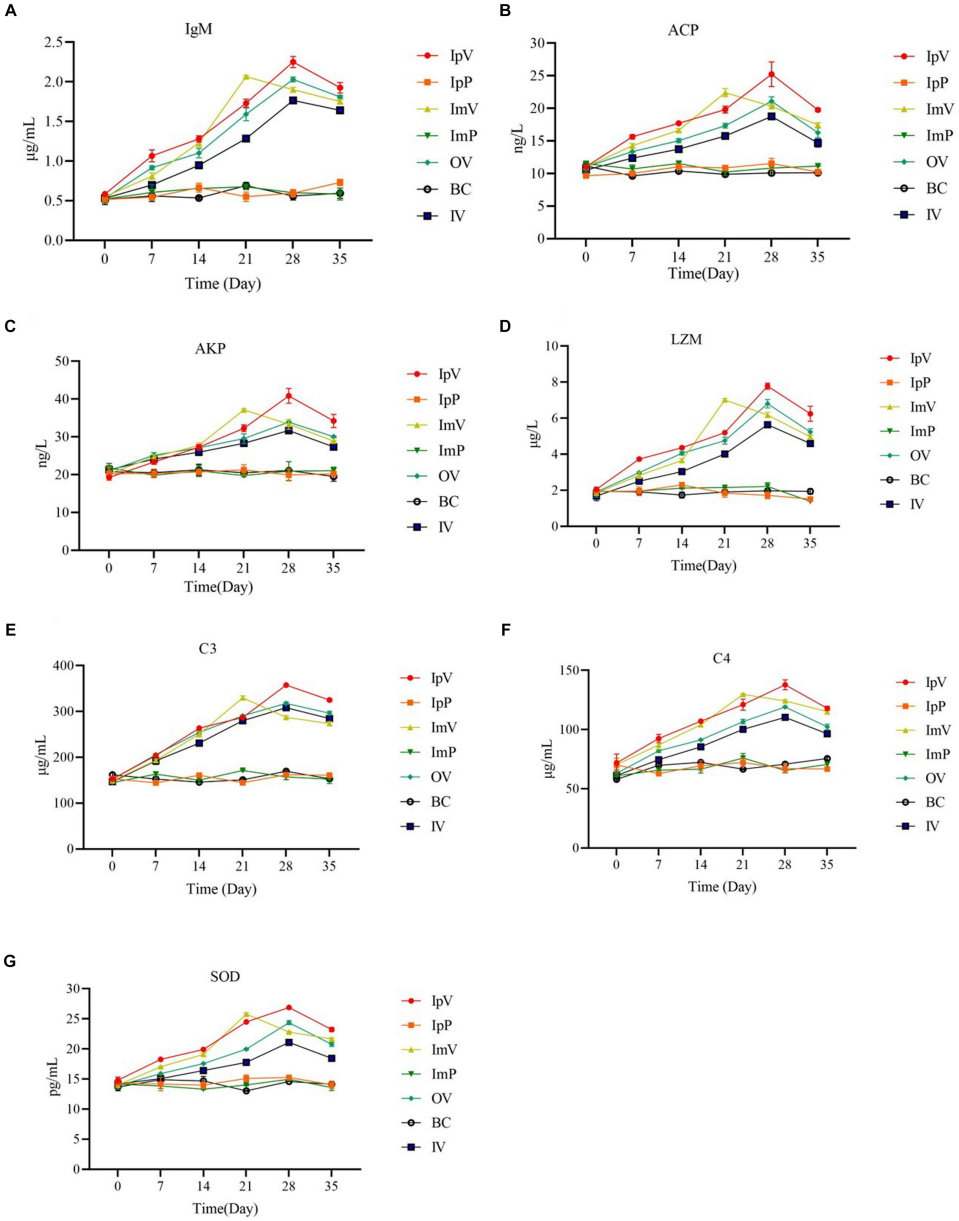
Detection results of the content of IgM **(A)**, ACP **(B)**, AKP **(C)**, LZM **(D)**; complement C3 **(E)**, complement C4 **(F)**, and SOD **(G)** in serum.

After immunizing *Carassius auratus* with the vaccine strain *ΔhisJ*, non-specific immune related molecules in the serum of the *Carassius auratus* were detected, including IgM, ACP, AKP, LZM; complement C3, complement C4, and SOD. Taking IgM as an example, the results (Figure 3A) show that the IgM content in the serum of *Carassius auratus* in various vaccine-immunized groups generally showed an upward trend after the primary immunization, and continued to increase after the secondary immunization. The IgM content in the serum of *Carassius auratus* in the IpV group, OV group, and IV group showed a continuous upward trend from the start of the initial immunization, reaching their respective peaks on the 28th d, then the IgM content began to decline, and by the 35th day after immunization, compared to the control group, the IgM content in the serum of the *Carassius auratus* in each immunized group was still significantly increased (*p* < 0.001). The IgM content in the ImV group showed a continuous upward trend from the 0 day of the primary immunization until the 21st d, reaching a peak, and then showed a downward trend. By the 35th d post immunization, compared to the control group, the IgM content in the immunized groups of *Carassius auratus* significantly increased (*p* < 0.001). It is worth noting that, on the 28th and 35th d post immunization, the non-specific immune molecules in the IpV group were higher compared to the other three vaccine immunization groups, suggesting that the intraperitoneal injection immunization route stimulates *Carassius auratus* to produce a more powerful non-specific immune effect. The BC group, IpP group, and ImP group showed no obvious trends in IgM content during each immunization period (*p* > 0.0.05), and there was little difference in IgM content in the serum between the groups. The trends in the levels of ACP, AKP, LZM, complement C3, complement C4, and SOD in the serum were the same as those of IgM.

### 3.5 Detection of the specific antibody against *Aeromonas veronii* in serum

**Figure 4:**
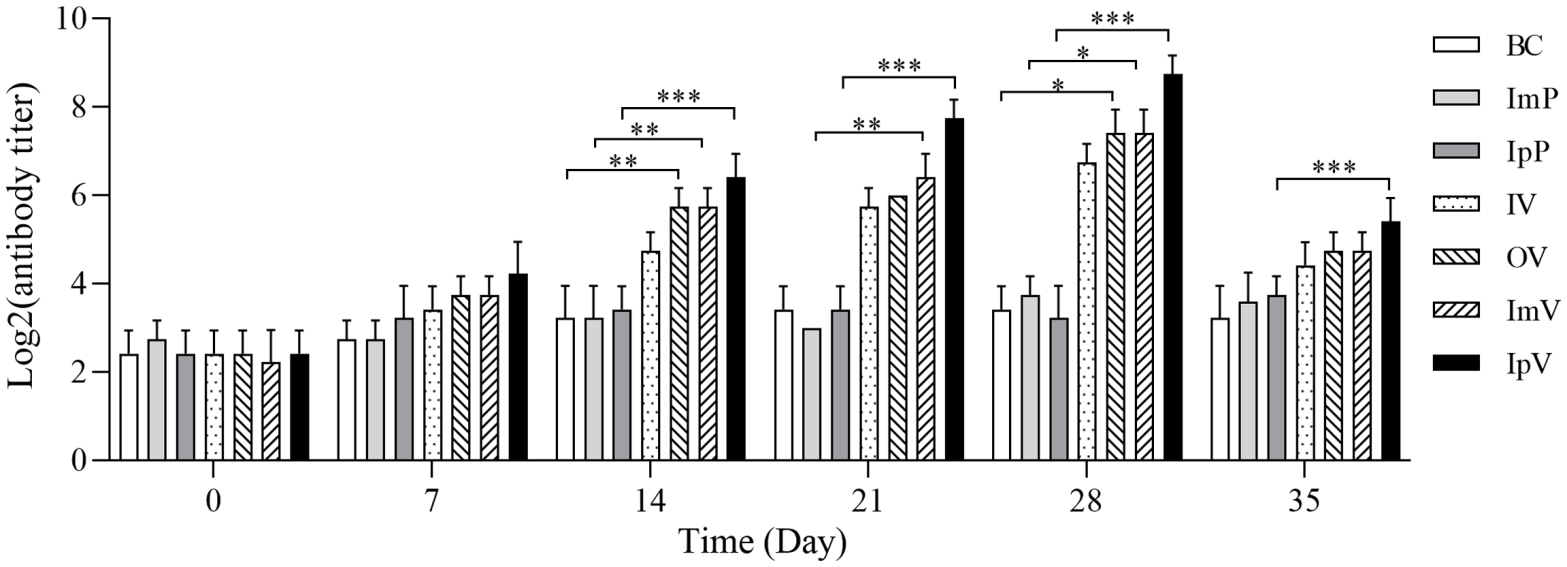
Detection results of the specific antibodies against *A.veronii* in serum.

Using the 96-well hemagglutination assay, the specific antibody titers of *Carassius auratus* serum immunized via different routes were tested. The results showed (Figure 4) that there were no significant differences in serum titers among the control groups (BC group, ImP group, and IpP group). The specific antibody titers of the *Carassius auratus* in each group immunized via different routes (IpV group, ImV group, IV and OV groups) were all higher than those of the control groups. Interestingly, the serum titer gradually increased with the increase of immunization times and the extension of time. On the 28th d, the serum antibody titer of the vaccinated *Carassius auratus* reached its peak and then declined. Among these, the serum antibody titers of the *Carassius auratus* in the IpV group were always the highest on the 7th, 14th, 21th, and 28th d post immunization. The antibody titers in both the OV and ImV groups rank second in terms of magnitude, with the group receiving immersion immunization displaying the lowest antibody titers. The results indicated that all four vaccine immunization routes could stimulate *Carassius auratus* to produce specific humoral immunity, but the intraperitoneal injection route of vaccination had the best immunization effect.

### 3.6 Results of white blood cell phagocytosis activity test

**Figure 5:**
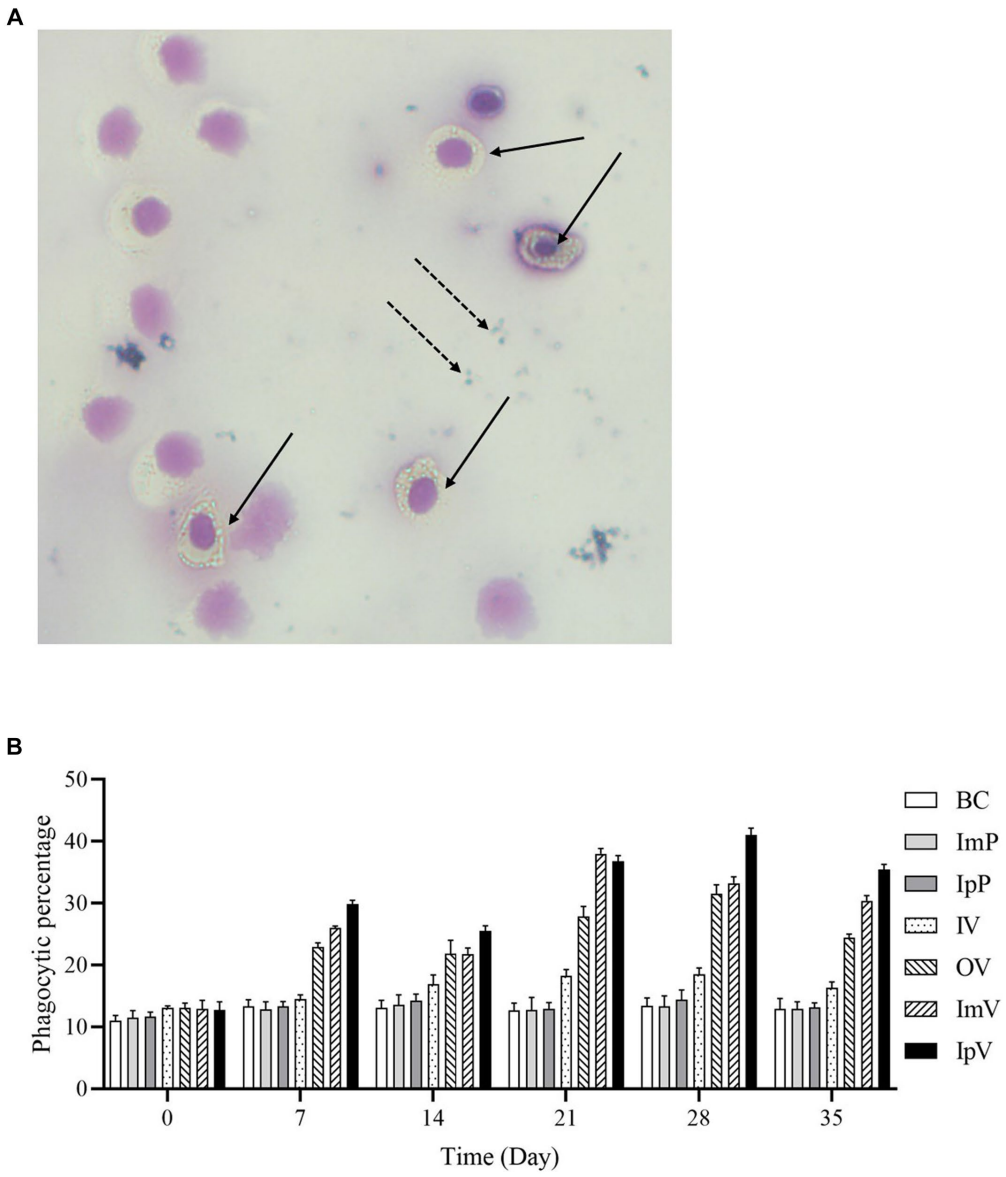
Leukocyte phagocytosis experiment. **(A)** Microscopic view of leukocyte phagocytosis (×400). Solid arrows indicate leukocytes engulfing *S.aureus*, while dashed arrows point to *S.aureus* not engulfed and located outside the cells. **(B)** Leukocyte phagocytosis ratio.

The anticoagulated blood of *Carassius auratus* post-vaccine immunization was taken and *Staphylococcus aureus CMCC(B)26,003* was added for a white blood cell phagocytosis experiment. The phagocytosis of 100 randomly observed white blood cells under a microscope was counted. The image of white blood cells engulfing bacteria is shown in Figure 5A, and the phagocytic ratio is displayed in Figure 5B. On the 0 d, the phagocytic ratio ranged between 10.01–13.73%, with no significant differences among the seven groups including the control group. However, following the vaccination of the vaccine test groups, from the 7th d of immunization, there was a significant upward trend in the phagocytic ratio of each vaccine immunization group, followed by a slight decrease on the 14th d. With the secondary immunization, the white blood cell phagocytic ratio continued to rise, reaching the peak phagocytic percentage for each vaccine test group on the 21st or 28th d of immunization, followed by a declining trend. On the 21st d post immunization, the white blood cell phagocytic percentage of the muscle ImV group increased significantly compared to the ImP group (*p* < 0.001); on the 28th d, the white blood cell phagocytic percentage of the IpV group increased significantly compared to the IpP group (*p* < 0.001); on the 28th d, the white blood cell phagocytic percentage of the oral vaccine group (OV) significantly increased compared to the BC group (*p* < 0.001); the white blood cell phagocytic percentage of the IV group showed an upward trend, but only on the 21st and 28th d post immunization, the phagocytic percentage was significantly different from the BC group (*p* < 0.05), with no significant differences at other time points. The white blood cell phagocytic ratios of the ImV and IpV groups were higher than those of the IV and OV groups, and the IpV group was higher than the ImV group. These results indicate that the intraperitoneal injection immunization route induced a more intense immune response.

### 3.7 Vaccination enhanced the expression of cytokines in different organs of *Carassius auratus*

**Figure 6:**
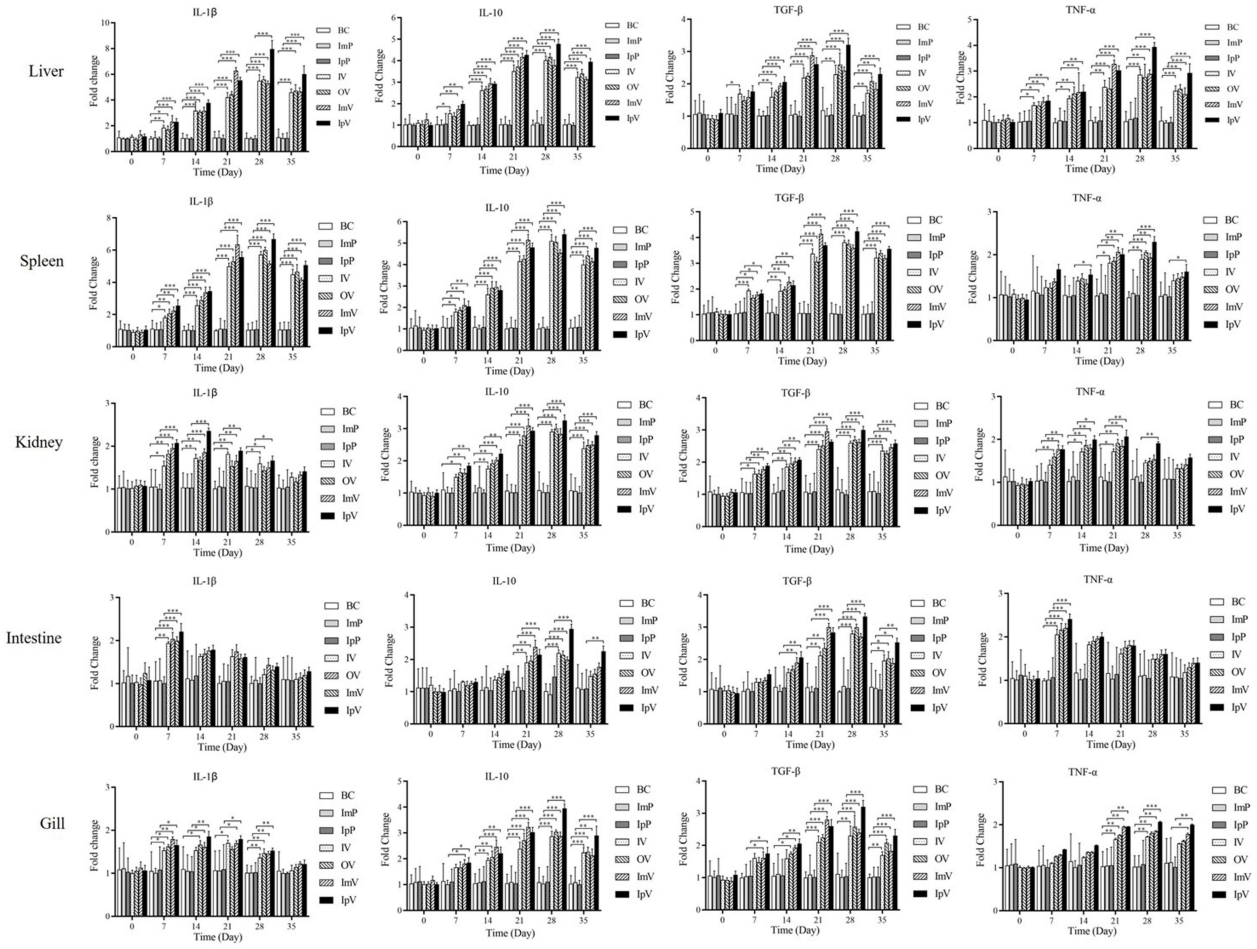
Analysis of cytokine expression in the liver, spleen, kidney, intestine, and gill of *Carassius auratus* following vaccine immunization.

Before immunization (on the 0 d), and on the 7th, 14th, 21st, 28th, and 35th d after the first vaccine immunization, *Carassius auratus* were randomly selected from each group for dissection. The liver, spleen, kidney, intestine, and gills were harvested to extract RNA, and qRT-PCR was performed to analyze the relative expression levels of cytokines IL-1β, TNF-α, IL-10, and TGF-β. The expression levels of the four cytokines in the liver were tested and analyzed. The results showed that (Figure 6), at all the testing days except the 0 d, the expression levels of IL-1β and TNF-α in the OV and IV groups were higher than those in the BC group. The highest levels in the OV, IV, and IpV groups were reached on the 28th d post-immunization, while for the ImV group, the peak was on the 21st d, followed by a decline in expression levels. The expression levels of cytokines in the spleen, kidney, intestine, and gills showed the same trends as those in the liver. Interestingly, the highest expression levels of the four cytokines in different organs of fish in the IpV group (on the 28th d) were higher than those in the other three vaccine immunization groups. The highest expression levels of the four cytokines in the ImV group fish (on the 21st d post-immunization) were higher than those in the OV and IV groups but still lower than those in the IpV group on the 28th d.

### 3.8 Challenge protection test

**Figure 7:**
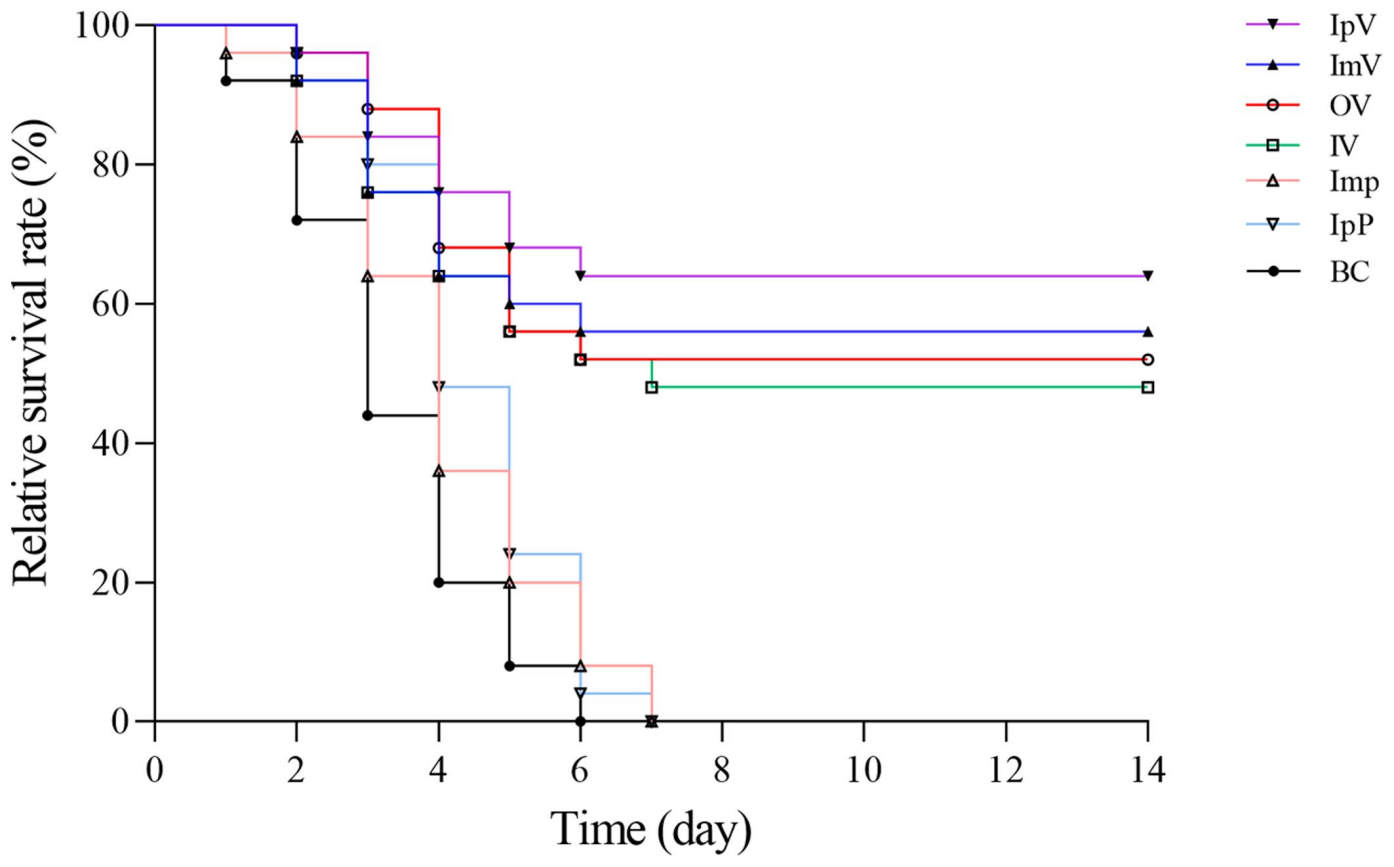
Relative survival rate after challenge with the virulent srain *A.veronii TH0426.*

The *Carassius auratus* in each group were intraperitoneally injected with the wild strain *A.veronii TH0426* on the 35th d after immunization to measure the immune protection effect. The *Carassius auratus* in the IpP, ImP and BC group all died within 6–7 ds after the challenge, exhibiting typical characteristics such as scale loss, bleeding, and abdominal swelling. Some fish in the various vaccine immunization groups also died within 6–7 ds after the challenge. As shown in Figure 7, On the 14th d after the challenge, the survival rate of the fish was 64% in the IpV group, 56% in the ImV group, 52% in the OV group, and 48% in the IV group. The results indicate that *Carassius auratus* immunized with the vaccine strain *ΔhisJ* have a certain protective effect against *A.veronii* infection. The protection rates vary with different immunization routes. The most effective immunization route is intraperitoneal injection, followed by muscle injection, oral immunization, and soaking immunization, in decreasing order of protection effectiveness.

### 3.9 Bacterial load in tissues after challenge with the wild strain *Aeromonas veronii TH0426*

**Figure 8:**
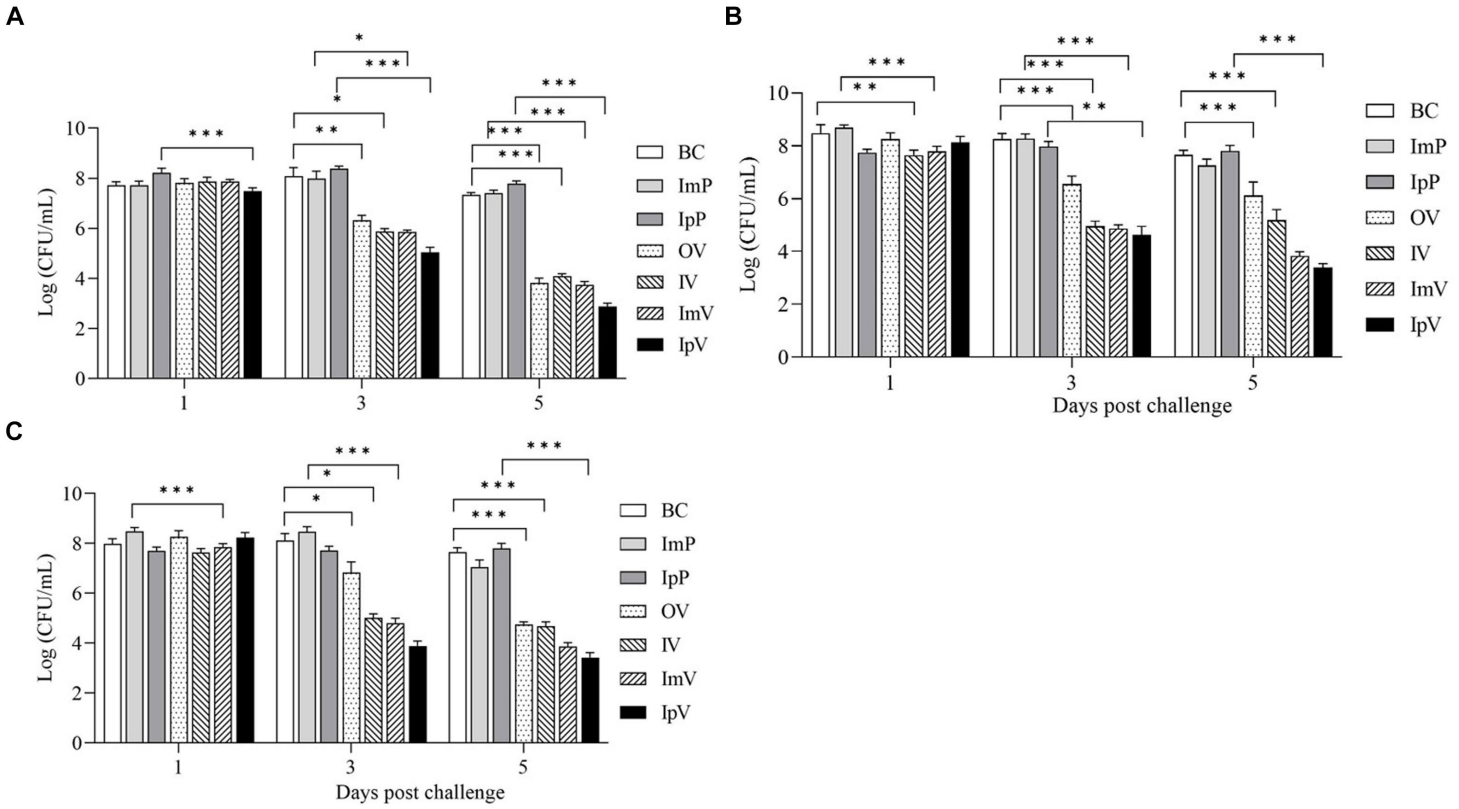
Bacterial load of *Carassius auratus* organs after challengement. **(A)** Liver, **(B)** spleen, and **(C)** kidney.

After intraperitoneal injection with the wild strain *A.veronii TH0426* in each immunized and control group of *Carassius auratus*, the liver, spleen, and kidney of dying fish were collected on the 1st, 3th, and 5th d post-challenge to measure the bacterial load in these organs. The results showed that (Figure 8) on the 1st day post-challenge, there was no significant difference in bacterial load compared to the respective control groups. However, on the 3th and 5th d post-challenge, the bacterial load in the organs of fish in the immunized groups was generally lower than that in their respective control groups. Additionally, the bacterial load on the 5th d was lower than on the 3th d, indicating a decreasing trend in bacterial load over time in the immunized groups. In contrast, the bacterial load in the liver, spleen, and kidney of the control group fish showed a trend of increase or maintained a high level without decreasing. Since the control group fish, lacking protective immunity, all died within 7 ds post-challenge, measuring bacterial load in their organ tissues on the 7th d was not feasible; hence, further analysis of the load was not conducted. The results of the bacterial load tests indicate that the vaccine strain *ΔhisJ* could gradually restrict the rapid proliferation of bacteria in the organ tissues of *Carassius auratus*, reducing the bacterial load in these tissues, thereby providing a certain level of immune protection against *A.veronii*. Notably, there were significant differences in the bacterial load in the organs of *Carassius auratus* immunized via different routes with the vaccine strain *ΔhisJ*. Fish vaccinated via intraperitoneal injection had the lowest bacterial load in their organs on the 3th and 5th d post-challenge, followed by those in the ImV group, the OV group, and the IV group.

## 4 Discussion

*Aeromonas veronii* is a significant aquatic animal pathogen that poses a great threat to aquaculture (5, 8). Vaccines are an essential means of preventing infection, and their development is of significant practical importance in aquaculture (22, 23). In China and throughout East Asia, where *Carassius auratus* farming is extensive, *A.veronii* infections severely jeopardize the healthful cultivation of these fish, necessitating public attention to vaccine development. In a previous study, a *hisJ* gene-deleted strain of *A.veronii TH0426* (namely *ΔhisJ*) was constructed and tested for immune protective effects in *Loaches*, achieving a survival rate of 65.66%, indicating its potential as a live attenuated vaccine (16). However, its efficacy in *Carassius auratus* remain unclear. Fish vaccines can be administered through injection (intraperitoneal and intramuscular), oral, and immersion routes, each eliciting different immune responses due to the recognition of vaccine antigens by different immune-presenting cells under varying immunization routes. Therefore, it is necessary to study the immune protective effects of *ΔhisJ* on *Carassius auratus* using different immunization routes, to evaluate the vaccine’s feasibility in these fish. This study demonstrated good genetic stability of *ΔhisJ* through passage experiments. Administering the vaccine to *Carassius auratus* using safe doses by intraperitoneal injection, intramuscular injection, oral immunization, and immersion immunization did not cause severe damage to the fish, indicating good safety of these four immunization methods. Blood immune index tests and challenge protection experiments showed that all four immunization methods provided good immune protection effect, with the effectiveness decreasing in the following order: intraperitoneal injection, intramuscular injection, oral, and immersion vaccination. These results lay an important foundation for the vaccine’s development.

Live attenuated vaccines can provide protection against infection in the host and prevent the spread of pathogens. However, the development of high-quality live attenuated vaccines involves three aspects: genetic stability, low virulence, and effective immune protection. Genetic stability is a critical indicator of vaccine quality (23, 24). To ensure the genetic stability of the BCG vaccine, it is stipulated that no more than 12 generations of seed strains be used in its production (25). Hence, to ensure vaccine quality, it is necessary to study and monitor the vaccine’s stability. Continued passage of vaccine strains, analyzed using techniques such as phenotypic analyses, DNA sequencing, PCR, DNA microarrays, complete genome sequencing, SNP detection, and macrorestriction digest of the genome, can reveal the genetic stability of the vaccine (25, 26). *In vitro* passage and PCR analysis was conducted in this study, and after 45 generations, the vaccine strain *ΔhisJ* remained a *hisJ*-deleted strain, indicating a good genetic stability. It is similarly to findings in vaccine studies of *Bordetella pertussis* and *Salmonella*, where the respective vaccines maintained their gene-deleted status after continuous passage, demonstrating good genetic stability.

The second criterion for live attenuated vaccines is to be entirely non-virulent for humans and animals (27, 28). Although previous study about the *Loach* had demonstrated that the vaccine strain *ΔhisJ* could establish short-term colonization or transient infection and was quickly cleared by the host, its virulence in *Carassius auratus* had not been determined. This study found that under the four immunization routes, the LD_50_ of the vaccine strain *ΔhisJ* in *Carassius auratus* was significantly lower than that of the parental strain *A.veronii TH0426*, and it did not cause severe pathological changes, indicating its safety in *Carassius auratus*. It’s worth mentioning that fish in the IpV and ImV groups exhibited strong stress reactions.

The live attenuated vaccines developed through genetic engineering techniques must not only ensure the safety of the vaccine strain for humans and animals but also exhibit strong and lifelong immunogenicity (28, 29). They should prevent infection in vital organs, reduce colonization in the host, and be effective against virulent strains. Strains that completely lose virulence are often quickly cleared by the host’s immune system, making it difficult to effectively activate an effective immune response (30). This research evaluated immune protective effects of the vaccine strain *ΔhisJ* across four immunization routes. The evaluation involved analyzing serum immune indices, white blood cell phagocytic capabilities, and the expression of immune genes in various organs of *Carassius auratus*. Additionally, the study assessed the survival rate of *Carassius auratus* during the virulent challenge and monitored changes in bacterial loads across different organs.

Non-specific immunity plays a pivotal role in the primary defense mechanism against diverse pathogenic invasions. In serum, key non-specific immune indicators comprise IgM, ACP, AKP, LZM, complement C3, complement C4, and SOD. This study demonstrated that immunization of *Crucian carp* through various routes leads to differential increases in these immune indicators (31, 32). Notably, the concentration of immune indicates of IpV group reached peak values on the 28th d after the first immunization, with levels slightly higher than them of other groups, while the concentration of immune indicates of ImV group peaked on the 21st d post-initial immunization, albeit slightly lower than the intraperitoneal group. The concentration of immune indicates of OV and IV groups also had higher levels of these immune indicators compared to the control group. Additionally, using agglutination tests to detect specific antibodies in serum, it was found that antibody titers in all immunization groups peaked on the 28th d after vaccination and then gradually declined. The intraperitoneal vaccination group had higher antibody titers than other groups, with no significant differences between the oral and intramuscular vaccination groups, and the lowest titers in the immersion vaccination group. The white blood cell phagocytosis test, an important indicator for measuring the effectiveness of vaccine immunization, revealed that the phagocytic ratio of white blood cells in all immunized groups continued to rise after the 21st d and peaked, with the concentration of immune indicates of ImV group peaking earlier than them of the others. IL-1β and TNF-α are pro-inflammatory factors in the body, which can limit the spread of pathogens, clear them, and activate the immune system, while IL-10 and TGF-β are anti-inflammatory factors, which can inhibit the excessive production of pro-inflammatory factors and prevent inflammatory damage to the body. This study found that after immunizing *Carassius auratus* with different immune routes, the expression levels of various cytokines in liver, spleen, kidney, intestine, and gill tissues increased to varying degrees and then declined. In the experiments by Zhang et al., the live attenuated vaccine *ΔhisJ* immunized *Loaches* by immersion and intraperitoneal injection, with anti-inflammatory (IL-10 and TGF-β) and pro-inflammatory (IL-1β and TNF-α) factors in the liver and spleen of *Loaches* peaking and then stabilizing within 28 ds post-immunization, which is not entirely consistent with our findings. In the experiments by Ma et al., a constructed *Streptococcus* DNA vaccine combined with the polymer PLGA to prepare an oral vaccine for *Tilapia* showed that the expression levels of pro-inflammatory cytokine genes such as IL-1β and TNF-α in the spleen and kidney tissues of Tilapia increased significantly and then decreased, similarly to the results of the oral vaccine group with *Carassius auratus* (33). In summary, these results show that using the vaccine strain *ΔhisJ* and immunizing *Carassius auratus* through different routes activated both non-specific and specific immune responses, with response intensity from high to low in the order of intraperitoneal injection, intramuscular injection, oral vaccination, and immersion vaccination.

By comparing the survival rates of individuals vaccinated or not vaccinated, the effectiveness of immunization interventions can be directly observed. In this study, *Carassius auratus* were vaccinated with a live attenuated vaccine through four different immunization routes and then challenged with a virulent strain. The results showed a relative survival rate of 64% in the IpV group, 56% in the ImV group, 52% in the OV group, and 48% in the IV group, indicating that different vaccination routes can provide varying degrees of protection to *Carassius auratus*. Although the injection immunization routes showed the highest protection rates compared to immersion and oral routes, both intramuscular and intraperitoneal injections caused varying degrees of injury to the fish, potentially leading to inflammation at the injection site and secondary bacterial infections (18, 20, 34). The research indicates that oral and immersion immunization methods are less effective, but they cause relatively less stress and injury to the fish. To address the suboptimal immune protective effect of these methods, various strategies can be employed. For immersion immunization, extending immersion time and increasing the concentration of bacterial suspension could enhance antigen uptake. For oral routes, selecting better vaccine encapsulating materials (such as alginate, chitosan, nano, and microparticles) or optimizing the vaccine preparation process can increase vaccine encapsulation rates and prolong the release of the vaccine in the gut, thereby enhancing the fish’s immune response. This study also measured the bacterial load in various organs of *Carassius auratus* post-challenge, finding that different immunization routes significantly reduced bacterial load in organs like the liver, spleen, and kidneys. The results suggest that the vaccine can limit the rapid proliferation of bacteria in the tissues of *Carassius auratus*, potentially helping to alleviate bacterial enteritis and septicemia. This also explains why the vaccinated *Carassius auratus* gradually stop dying after 7 ds post-immunization.

In conclusion, this study demonstrates that the vaccine *ΔhisJ* can be used as a live attenuated vaccine for *Carassius auratus*, with good genetic stability and safety. Vaccination through different routes can enhance the immune level of *Carassius auratus* and provide certain protection against *A.veronii* infection. It is particularly noteworthy that, although the immune protective effect of intraperitoneal and intramuscular injection routes is superior to oral and immersion immunization, the injection routes may cause injury to the fish, with oral immunization routes offering better protection than immersion. This research lays a significant foundation for the development of live attenuated vaccines of *A.veronii*, but more attention needs to be paid to issues such as genetic variation in live attenuated vaccines. Nevertheless, this study possesses inherent limitations. A more extensive scale of experimental animals is essential to thoroughly assess vaccine safety and eliminate possible toxic effects. Moreover, evaluating vaccine efficacy must entail quantifying the duration of immune protection, given its critical role as an efficacy indicator. Addressing these issues is pivotal for advancing the vaccine’s development and application in *Carassius auratus*.

## 5 Conclusion

The *hisJ* gene-deleted strain of *A.veronii*, as a live attenuated vaccine, is safe for *Carassius auratus* when administered via intramuscular injection, intraperitoneal injection, oral, and immersion methods. Immunizing *Carassius auratus* through different routes can enhance the phagocytic activity of phagocytes in their serum, increase the activity of non-specific immune-related enzymes, and raise the level of specific serum antibodies and cytokines in the fish. The survival rate of *Carassius auratus* after being challenged with a virulent strain shows variation depending on the route of vaccination with the vaccine strain *ΔhisJ*. Furthermore, post-vaccination, the spread of *A.veronii* in the organ tissues can be restricted.

## Data availability statement

The datasets presented in this study can be found in online repositories. The names of the repository/repositories and accession number(s) can be found in the article/supplementary material.

## Ethics statement

Experimental Animal Regulation Ordinances defined by Hebei Provincial Department of Science and Technology (HPDST2020-17) and Regulations for Animal Experimentation at Jilin Agricultural University (JLAU08201409). The study was conducted in accordance with the local legislation and institutional requirements.

## Author contributions

TW: Conceptualization, Investigation, Methodology, Writing – original draft, Writing – review & editing. RM: Data curation, Methodology, Project administration, Software, Writing – review & editing. XP: Conceptualization, Investigation, Writing – original draft, Writing – review & editing. FW: Data curation, Formal analysis, Methodology, Writing – original draft. ZZ: Formal analysis, Project administration, Validation, Writing – review & editing. QS: Funding acquisition, Project administration, Resources, Writing – review & editing. XS: Conceptualization, Funding acquisition, Investigation, Resources, Writing – original draft, Writing – review & editing. GG: Funding acquisition, Project administration, Writing – review & editing.
